# Some Differences in *Some*: Examining Variability in the Interpretation of Scalars Using Latent Class Analysis

**DOI:** 10.5334/pb.bc

**Published:** 2015-03-13

**Authors:** Tom Heyman, Walter Schaeken

**Affiliations:** 1 University of Leuven, Department of Experimental Psychology, Leuven, Belgium

**Keywords:** scalar inferences, latent class analysis, interindividual differences

## Abstract

The present study investigated people’s understanding of underinformative sentences like ‘Some oaks are trees’. Specifically, the scalar term ‘some’ can be interpreted pragmatically, Not all oaks are trees, or logically, some and possibly all oaks are trees. The aim of this study was to capture the interindividual variability in the interpretation of such sentences. In two experiments, participants provided truth value judgments for 20 underinformative sentences on which a latent class analysis was performed. The results revealed three latent classes: a consistent pragmatic group, a consistent logical group and an inconsistent group. Furthermore, we examined whether this interindividual variability could be explained by text characteristics, response times, cognitive abilities and personality traits. The results showed that only participants’ response times to the underinformative sentences could predict class membership. Specifically, the slower participants responded, the more likely they were to interpret underinformative sentences consistently pragmatic or inconsistent instead of consistently logical.

## Introduction

Consider the following utterance from a professor in statistics:

(1) Some students dislike statistics.

Most people interpret *some students* as *not all students* (i.e., the pragmatic interpretation), though logically it means *some and possibly all students*. This tendency finds its roots in the assumption that people make their contribution to a conversation as informative as possible (Grice, 1975). The rationale is that the professor would have used a stronger, more informative term if he knew that all of his students disliked statistics, thus *some* is interpreted as *not all*. This scalar inference, as it is called, has been studied extensively and different theories have been developed to explain the underlying processes (see Noveck & Reboul, 2008, for an overview). In particular, the contradiction between two popular views on scalar inferences, default theories and contextual accounts, is what fuelled much research on this topic. Default theories assume that scalar inferences arise automatically without much cognitive effort. A pragmatic understanding of *some* is the default interpretation, but it may be cancelled in certain contexts (Chierchia, 2004; Levinson, 2000). Contextual accounts like relevance theory on the other hand posit that the pragmatic interpretation requires processing time, hence it is only endorsed when it yields sufficient positive cognitive effects (Sperber & Wilson, 1986, 1995).

Of particular interest are underinformative utterances like:

(2) Some oaks are trees.

A pragmatic understanding of (2) implies that the sentence is false, since all oaks are in fact trees. On the other hand, the sentence is true when one holds a logical interpretation (i.e., some and possibly all oaks are trees). The double truth value of underinformative sentences has been exploited in several experimental studies to pit default theories against contextual accounts. Seminal work from Bott and Noveck (2004) showed that participants in a sentence verification task are slower when they endorse the pragmatic interpretation (i.e., judging a sentence like (2) as false) than when they endorse the logical interpretation (i.e., judging (2) as true). Building on this study, Bott, Bailey, and Grodner (2012) reported experiments suggesting that the scalar inference itself is cognitively costly, thereby ruling out that the effects observed by Bott and Noveck were purely the result of verification processes. These results, together with the observation that scalar inferences are context dependent (e.g., Bonnefon, Feeney, & Villejoubert, 2009), provide evidence against a default account of scalar implicatures.

Since the findings from Bott and Noveck (2004), several new accounts (or modifications of older ones) have been proposed (Breheny, Ferguson, & Katsos, 2013; Chiercia, Fox, & Spector, 2008; Degen & Tanenhaus, 2011, 2014; Huang & Snedeker, 2009). Of particular interest to the present paper are the one-step and two-step models. Two-step models assume that the literal meaning of *some* is derived first after which the enriched *not all* inference is drawn (Huang & Snedeker, 2009; Tomlinson, Bailey, & Bott, 2013). In a way, a two-step model can be viewed as the mirror-image of a default theory. Where the latter posits that the *not all* interpretation is the default that can be overruled, two-step models consider the literal interpretation to be default, which serves as a stepping stone to derive the scalar inference. In contrast, the constraint-based one-step model (Degen & Tanenhaus, 2011; 2014) rejects the notion that the literal interpretation is somehow privileged. Instead, it postulates that the appropriateness of a certain interpretation is assessed by integrating multiple contextual cues. The outcome of this process then determines whether or not the scalar inference will be derived.

Despite the general tendency to interpret *some* as *not all*, theories are largely silent when it comes to interindividual differences. That is, research shows that people vary in how they interpret underinformative utterances (for an overview see Dieussaert, Verkerk, Gillard, & Schaeken, 2011). Furthermore, it has been argued that people differ considerably in the consistency with which they interpret underinformative sentences. That is to say, some people interpret such sentences with great consistency, while others often switch between a pragmatic and a logical interpretation. The aim of the present study was to uncover such individual differences using latent class analysis (McCutcheon, 1987).

Latent class analysis is a statistical technique to classify observations into a number of categories. It is based on the premise that a number of observed variables (e.g., several medical symptoms like fever, a cough,…) covary because of their relation with an unobserved underlying variable (e.g., a disease like the flu). With respect to scalar implicatures, we asked participants to evaluate the truth value of 20 underinformative sentences like (2). The responses of participants (i.e., true or false) to the different items are the manifest variables for the latent class analysis. The rationale of the analysis is that one latent variable, which we will call interpretation mode, underlies the 20 covarying response vectors. Interpretation mode here indicates the way in which participants understand underinformative sentences. Based on previous studies one could discern at least two modes: a “logical mode” and a “pragmatic mode”. That is to say, some participants exhibit a clear preference for a logical interpretation of the scalar term *some* (i.e., the logical mode) whereas others endorse a pragmatic interpretation (i.e., the pragmatic mode). So when performing a latent class analysis, one would expect a solution that comprises at least two latent classes. In addition, Dieussaert and colleagues (2011) claim that there are actually three groups: consistently logical participants, consistently pragmatic participants and inconsistent participants. The latter are those who (often) switch between a logical and a pragmatic interpretation. However, the boundary between consistent and inconsistent is rather vague. Dieussaert and colleagues (2011) classified participants as inconsistent if their modal answer was given in less than 90% of the trials. One advantage of latent class analysis is that it obviates the need to use such relatively arbitrary criteria to classify participants into categories. It estimates the exact number of latent classes using model selection measures. In this way, one can evaluate whether there is evidence for a third class (i.e., the inconsistent participants) or even for four classes. Moreover, latent class analysis allows for uncertainty in the classification of participants, such that categorization is not an all or none matter.

So the first goal of the study was to capture interindividual differences in the way people interpret scalar implicatures. A second goal was to examine whether we could predict these differences. Previous research using underinformative sentences has shown that a pragmatic interpretation requires more processing time than a logical interpretation (Bott et al., 2012; Bott & Noveck, 2004; Tomlinson et al., 2013). Based on these findings we investigated in Experiment 1 whether participants’ response times were related to their class membership, such that slow participants were more likely to be categorized as pragmatic participants than as being logical. Secondly, we manipulated the font in which sentences were written because several studies provided evidence for the claim that a hard to read font triggers more elaborate reasoning processes (Alter & Oppenheimer, 2008; Alter, Oppenheimer, Epley & Eyre, 2007). Specifically, participants presented with the Cognitive Reflection Test (henceforth CRT, Frederick, 2005) in a hard to read font, tended to override their intuitive default more frequently than participants in the regular font condition (Alter et al., 2007). The present study sought to examine whether this would also apply to scalar inferences. Particularly, the different theories discussed above vary in their view on how the *not all* interpretation arises. Default theories assume that the pragmatic interpretation arises automatically, whereas two-step models hypothesize that the logical interpretation is the default. If a hard to read font indeed prompts a cancellation of the default, two-step models would predict more pragmatic interpretations, where default theories would expect less pragmatic interpretations. It is less obvious what constraint-based one-step models would predict in this situation. The interpretation of the scalar term in this framework is argued to depend on cues in the linguistic and discourse context (Degen & Tanenhaus, 2014). It is highly questionable whether font would actually fit this definition of a cue and even if it does, it is not clear what effect it would have on the interpretation of the scalar term. If anything, the one-step model would expect the font manipulation to have no effect because it (presumably) does not influence the linguistic and discourse context.

In Experiment 1, we tested these predictions by assigning participants either to a normal font or a hard to read font condition. The question was whether participants in the latter condition were more, less, or equally likely to understand the underinformative sentences pragmatically. To examine the relationship between response time and font on the one hand and class membership on the other hand, we used an extension of the basic latent class analysis. Latent class regression allows one to add covariates to predict the probability of latent class membership (Dayton & Macready, 1988). So the question is whether response time and font are related to the interpretation mode of participants. In sum, the Experiment 1 aimed to a) uncover systematic interindividual differences in interpretation of scalar implicatures and b) relate these differences to two predictors, response time and font.

## Experiment 1

### Method

#### Participants

Participants were 139 first-year psychology students of the University of Leuven (24 men, 115 women, mean age 18 years), who participated in return for course credit. All participants were native Dutch speakers.

#### Materials

The stimulus material consisted of 20 underinformative sentences like *‘Some oaks are trees’* (i.e., ‘*Sommige eiken zijn bomen’* in Dutch) and 20 control sentences taken from De Neys and Schaeken (2007). Participants were required to assess the truth value of each sentence. Control sentences were always unambiguously true (e.g., ‘*Some insects are wasps’*; ‘*Sommige insecten zijn wespen’* in Dutch) or false (e.g., ‘*Some beetles are flowers’*; ‘*Sommige kevers zijn bloemen’* in Dutch). The font in which these sentences were presented, obtained from Alter and Oppenheimer (2008), was either 16 point Times New Roman (i.e., the normal condition) or 16 point italicized Haettenschweiler (i.e., the hard to read condition). All materials were in Dutch.

#### Procedure

Participants were randomly assigned to the normal or hard to read font condition^1^. The order of the 40 sentences was also random and varied over participants. Each sentence was presented in the center of a computer screen and participants indicated whether it was true or false by pressing “w” or “n” on an AZERTY keyboard. The sentence remained on the screen until a response was made. Four different control practice trials preceded the main experiment. The study has been approved by the medical ethics committee of the University of Leuven (reference number: ML8930).

### Results

The responses of participants to the 20 underinformative sentences were used as input to the latent class analysis. Participants with less than 75% of the control trials correct were removed from the analyses (N = 9). A model selection approach was taken to estimate the number of latent classes. That is to say, the fit of five latent class models, representing a one, two, three, four, or five class solution, was compared. This was accomplished via the poLCA R package (Linzer & Lewis, 2011). We ran each model 50 times to avoid local maxima. Model fit was assessed with the Bayesian Information Criterion or BIC-score. It compares the goodness of fit to the number of parameters of the model. Models with lower BIC-sores are preferable. The BIC-scores for the five models indicate that the three class model provides the best fit of the data (see Table 1).

**Table 1:** BIC-scores for the five latent class models. Models only differ in the number of latent classes they presume.

Models	BIC

One class model	3627
Two class model	2109
**Three class model**	**2057**
Four class model	2112
Five class model	2174

*Note*. The best fitting model is printed in bold.

To give a substantive interpretation of the three classes (preliminary denoted as class A, B and C), we looked at the class-conditional response probabilities. These estimate the probability of interpreting a certain underinformative sentence pragmatically (or logically) *given* class membership: when someone is a member of class A, what is then the probability to interpret ‘*Some oaks are trees’* pragmatically. The class-conditional probabilities were rather stable over items meaning that a member of, say, class A has a more or less equal probability to interpret ‘*Some oaks are trees’* pragmatically as to interpret ‘*Some ants are insects’* pragmatically. So based on these probabilities one can distil the meaning of the three classes. The average probability of a pragmatic interpretation for Class A, B and C was 0.94, 0.05 and 0.64. Hence, they were conceived, respectively, as a pragmatic class, a logical class and an inconsistent class. Thus, these findings suggest that there are three groups of participants corresponding to three interpretation modes: logical, pragmatic and inconsistent. As we already mentioned in the introduction, some uncertainty exists in the classification of participants into these groups. Nevertheless, one can predict class membership by modal assignment (i.e., assigning participants to their most probable class). This approach makes it possible to estimate the number of participants per class. It shows that the majority of the participants are consistent in their interpretation, 45% are pragmatic and 34% are logical, whereas 21% are inconsistent.

To test whether these interindividual differences can be explained by font and response time, we ran a latent class regression analysis which allows to add covariates to predict latent class membership. The three covariates here were Font, a dichotomous variable, Response Time Underinformative (RTU), which was the z-transformed median response time per participant over all underinformative items (*M* = 2022 ms, *SD* = 511) and Response Time Control (RTC), the z-transformed median response time per participant over all control items (*M* = 2092 ms, *SD* = 461). To assess the predictive value of these variables, we again opted for a model comparison approach (see Table 2).

**Table 2:** BIC-scores for the eight latent class regression models. Models presume three latent classes and differ only in the covariates that are included.

Models	BIC

Empty model (no covariates)	2057
Font	2063
**RTU**	**2040**
RTC	2058
Font + RTU	2046
Font + RTC	2063
RTU + RTC	2044
Font + RTU + RTC	2050

*Note*. The best fitting model is printed in bold.

**Figure 1:**
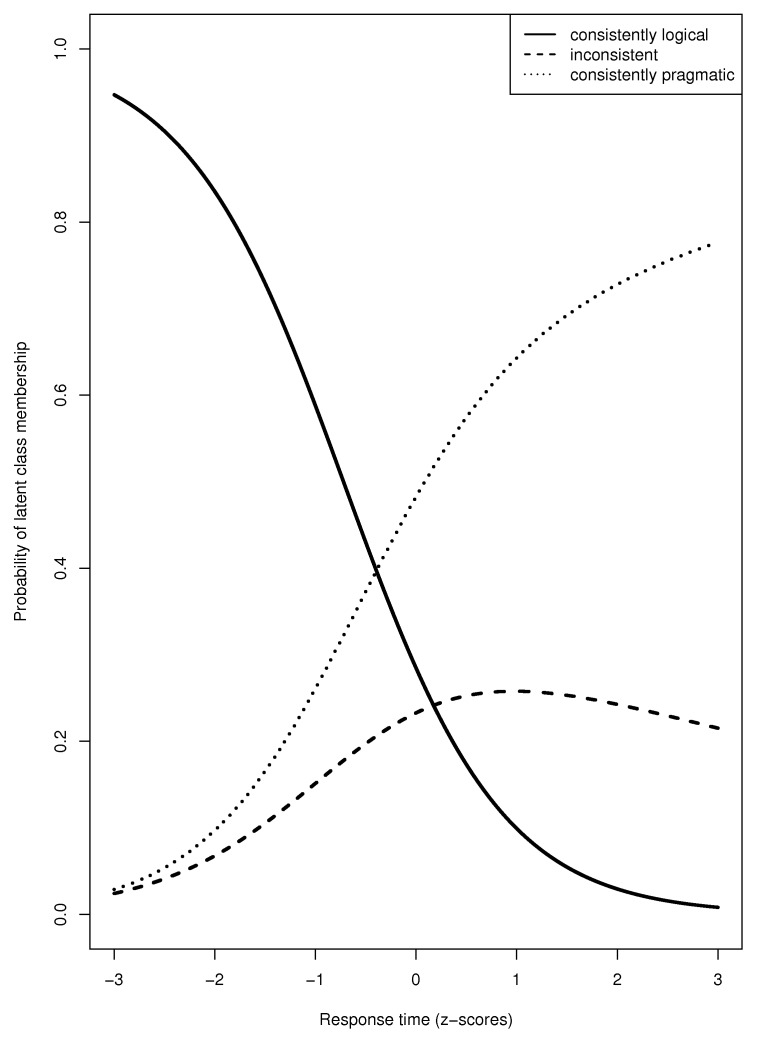
The probability of latent class membership for different response times. The x-axis represents the standardized median response times to the underinformative sentences. The y-axis denotes the predicted probability of latent class membership.

The preferred model according to the BIC-scores was the one with Response Time Underinformative as the only covariate. The models that included Font and/or Response Time Control yielded higher BIC-scores, which indicates that these variables were not significantly related to class membership. Taken together, we can predict the probability of latent class membership from the response times of participants to the underinformative sentences, but not on the basis of their response times to the control sentences nor on the font in which these sentences were presented. Specifically, the probability of belonging to the logical group relative to the pragmatic group *increases* when Response Time Underinformative *decreases* (*t*(66) = 3.47, *p* < .001). Similarly, the probability of belonging to the logical group relative to the inconsistent group also *increases* when Response Time Underinformative *decreases* (*t*(66) = 2.26, *p* = .03). There was no significant difference between the pragmatic class and the inconsistent class (*t*(66) = 0.44, *p* = .69). In other words, the faster a participant is, the higher the probability of interpreting underinformative sentences logically than pragmatically or inconsistently (see Figure 1). Importantly, this effect is not driven by reading speed in general since response times to the control sentences were unrelated to class membership.

### Discussion

Experiment 1 documents interindividual differences in the derivation of scalar inferences. Using a latent class analysis, it shows that there are three groups of people: those who consistently endorse a pragmatic interpretation (i.e., *some* means *not all*), those who consistently adopt a logical interpretation (i.e., *some* means *some and possibly all*) and those who are rather inconsistent. Furthermore, people’s class membership can be predicted by their response time to the underinformative sentences. Specifically, participants were more likely to be inconsistent or consistent pragmatic interpreters when their responses to underinformative sentences were relatively delayed, whereas participants who responded relatively fast to underinformative sentences were more likely to endorse a logical interpretation. These results conceptually replicate and extend the finding of Bott and colleagues (2004; 2012) that pragmatic interpretations require more time than logical interpretations. An alternative explanation of the relation between response time to underinformative sentences and interpretation mode is that some participants merely ignored the quantifier and evaluated the truth value of the embedded proposition (e.g., oaks are trees). Indeed, sentences were presented as a whole rather than word per word, which might foster such a strategy. Neglecting the quantifier would arguably lead to shorter response times and would elicit “yes” responses to underinformative sentences (e.g., oaks are in fact trees). However, the former would also hold for control sentences. That is, ignoring the quantifier would decrease response times *in general*. If this explanation were to be true, one would also expect a relation between response times to control sentences and the interpretation of underinformative sentences. The results revealed that this was not the case, which implies that interpretation mode is not linked to a general slowing of response speed. In addition, several (control) experiments have been carried out, using paradigms where sentences are presented word by word, which attributed the slowing observed for pragmatic interpretations to the computation of the scalar inference itself (Bott et al., 2012).

Besides response time we also examined the effect of font on class membership. Because a hard to read font has been shown to reduce reliance on defaults (Alter et al., 2007), we suspected that it might also have an influence on the interpretation of underinformative sentences. Specifically, default theories would expect more logical interpretations in the hard to read font condition, whereas two-step models would predict the opposite. However, the results showed no effect of font (i.e., 60% pragmatic responses in the hard font versus 57% in the normal font condition). This finding is more in line with the constraint-based one-step model of Degen and Tanenhaus (2014), which postulates that cues in the linguistic and discourse context determine the interpretation of the scalar term. Font size is definitely not a linguistic cue and is arguably not part of the discourse context. Hence, this model could account for the fact that font has no influence on the interpretation of underinformative sentences.

One alternative explanation for the lack of an effect may be that the manipulation failed or was not strong enough. Even though the same fonts were used as in Alter and Oppenheimer (2008), the hard to read font may have failed to trigger more elaborate reasoning processes. The data seem to support this hypothesis as participants in the hard to read font condition were not significantly slower than those in the normal font condition (*t*(128) = 1.24, *p* = .22)^2^. This result casts some doubt on the robustness of perceptual fluency manipulations. Moreover, it may even nuance the pervasiveness of the perceptual fluency *effect* per se: there is perhaps no such effect in sentence verification. The latter assertion appears to contradict with the results from Reber and Schwarz (1999), who reported that perceptual fluency affects truth judgments of statements like ‘*Osorno is in Chile*’. However, closer inspection of their results, using the Bayesian hypothesis test developed by Rouder et al. (2009), indicates that the null hypothesis (i.e., no difference between highly and moderately visible conditions) is actually about 5 times more likely than the alternative. Furthermore, Thompson and colleagues (2013) recently examined the effect of perceptual fluency on accuracy in a variety of cognitive tasks. They found no evidence that disfluency increases accuracy except in the CRT, where only the most cognitive able showed the effect.

In sum, the latent class analysis employed in Experiment 1 showed large interindividual differences in the interpretation of a scalar term like *some*. In a second experiment we sought to explain this variability by linking interpretation mode to a range of cognitive and personality traits. The included covariates from the cognitive domain were working memory capacity and the ability to override an intuitive default. As noted by De Neys and Schaeken (2007), the nature of the relation between working memory and the understanding of underinformative sentences has repercussions for the theories about scalar inferences. If an enriched interpretation requires cognitive resources as suggested by relevance theory and two-step models^3^, one might expect people with limited working memory capacity to endorse the pragmatic reading of *some* to a lesser extent (Dieussaert et al., 2011). However, results from previous studies are equivocal. Dieussaert and colleagues did not find a link between working memory span and the number of pragmatic responses, whereas Feeney, Scrafton, Duckworth and Handley (2004) obtained a *negative* correlation. One goal of Experiment 2 was to provide more insight on this issue.

A similar argument can be made for people’s ability to override a default. It is conceivable that people who rely heavily on heuristics may rarely move to a more elaborate understanding of a scalar term. In contrast, those who are able to inhibit their sometimes incorrect, gut feeling may also endorse a more cognitively demanding interpretation. The predictions are very similar to these for the font manipulation in Experiment 1. If the literal meaning of *some* is default (as assumed by two-step models) on might expect heuristic thinkers to more readily adopt a logical interpretation mode, whereas a default theory might expect the reverse. Again, it is not clear what one-step models would predict as they reject the notion of a default interpretation.

Besides the two cognitive variables described above, we also included ten personality covariates, which can be divided into two groups of five. One group consists of the subscales of the Autism-Spectrum Quotient questionnaire: Social skill, Communication, Imagination, Attention to detail, and Attention switching (Baron-Cohen, Wheel­wright, Skinner, Martin, & Clubley, 2001). The others are the so-called Big Five personality traits: Extraversion, Agreeableness, Conscientiousness, Neuroticism, and Openness to Experience (McCrae & John, 1992). The latter were included for exploratory purposes only. The former, however, were used to elaborate on findings from Nieuwland, Ditman, and Kuperberg (2010), who found that pragmatically skilled people (i.e., those with a low score on the Communication subscale) were more sensitive to underinformative statements. That is, Nieuwland and colleagues found a relation between N400, an ERP component associated with semantic processing, and people’s communicative abilities. This was interpreted as to mean that communicatively skilled people are more responsive to pragmatic violations as indexed by the N400 component. Experiment 2 examined whether these findings generalize to sentence verification data.

## Experiment 2

### Method

#### Participants

Participants were 322 first-year psychology students of the University of Leuven (57 men, 262 women, mean age 18 years), who participated in return for course credit. All participants were native Dutch speakers.

#### Materials

Working memory capacity was assessed using a Dutch, computerized, and group administrable adaptation of the Operation Span Test (henceforth GOSPAN, De Neys, d’Ydewalle, Schaeken, & Vos, 2002). In this test, participants have to judge the validity of a simple equation (e.g., is 4/2 – 1 = 5?) after which a to-be-remembered word is briefly presented. After two to six of such equation-word sequences, participants are prompted to write down the words in the correct order. Participants’ working memory scores tally the number of correctly remembered words, but only of the completely reproduced word series (so partially correct word series do not count towards the score). There are 15 series in total, which can maximally lead to a score of 60.

The ability to override an intuitive default was measured by the three-item CRT (Frederick, 2005). It comprises three open ended short questions such as *If it takes 5 machines 5 minutes to make 5 widgets, how long would it take 100 machines to make 100 widgets?* The intuitive answer to these questions (i.e., 100 for the example item) always differs from the correct answer (i.e., 5 for the example item). Participants’ score on the CRT is simply the number of correctly solved questions.

The Big Five dimensions (i.e., Extraversion, Agreeableness, Conscientiousness, Neuroti­cism, and Openness) were measured with the Dutch version of the Ten Item Personality Inventory (henceforth TIPI, Hofmans, Kuppens, & Allik, 2008). Participants had to indicate on a seven-point scale the extent to which ten pairs of traits like *extraverted, enthusiastic* applied to them. There is one pair for every pole of the Big Five dimensions and participants get a score per dimension by averaging over the two relevant items (after reverse scoring where appropriate).

The Dutch Autism-Spectrum Quotient questionnaire was also administered (henceforth AQ, Hoekstra, Bartels, Cath, & Boomsma, 2008). It consists of fifty statements and participants have to rate on a four-point scale to what extent they agree or disagree with each statement. The AQ questionnaire consists of five subscales (i.e., Social skill, Communication, Imagination, Attention to detail, and Attention switching) each comprising ten statements. Summing across the ten statements (again after reverse scoring when appropriate) yields a score on every subscale. The higher the score, the more autistic traits one possesses. Hence, people with a low score on the Communication scale, with items such as *I find it easy to “read between the lines”*, are more communicatively skilled.

Finally, the sentence verification task was the same as in Experiment 1, except that the items were always presented in a normal font.

#### Procedure

Participants completed the tasks in the following order: CRT, TIPI, AQ, sentence verification, and GOSPAN. All tasks were performed on a computer, except that the word series of the GOSPAN had to be written down on an answer sheet. The procedure of the sentence verification task was the same as in Experiment 1.

### Results

Participants who did not complete all tasks or whose accuracy on the control trials of the sentence verification task was below 75% were removed from the analyses (N = 20). The results of the latent class analysis replicated those of Experiment 1 in that the three class solution provided the best fit of the data (see Table 3). The classes can again be interpreted in terms of three interpretation modes: logical (18% of the participants), pragmatic (71%), and inconsistent (12%). Note that the pragmatic class appears to be larger than in Experiment 1, even though the same stimulus material was used. So even when variables such as proportion of fillers and language, which have been argued to influence the number of (consistent) pragmatic interpretations (Dieussaert et al., 2011), are held constant, there can be variability in the respective size of each latent class over experiments.

**Table 3:** BIC-scores for the five latent class models. Models only differ in the number of latent classes they presume.

Models	BIC

One class model	7188
Two class model	4536
**Three class model**	**4401**
Four class model	4431
Five class model	4503

*Note*. The best fitting model is printed in bold.

In a next step, a series of latent class regression analyses were performed. First, all models with a single main effect were fitted (see Table 4). The results indicate that neither the cognitive nor the personality covariates are able to predict class membership. Except for the model with CRT, which fits the data as well as the empty model (both BIC’s = 4401), all other models perform worse (all BIC’s ≥ 4407). A follow-up analysis examining the CRT model suggested that the higher people score on the CRT, the higher the probability of adopting a logical interpretation mode compared to an inconsistent interpretation mode (*t*(238) = 1.67, *p* = .10). Similarly, the probability of belonging to the pragmatic class relative to the inconsistent class also seems to increase as CRT-score increases (*t*(238) = 1.64, *p* = .10). However, there was no such effect for the logical versus the pragmatic class (*t*(238) = 0.44, *p* = .66). These findings may suggest that people with a high CRT-score are more persistent in their interpretation of scalar terms. Alternatively, some participants may have been more motivated to perform well on the various tasks, which caused them to obtain higher CRT-scores and adopt a consistent interpretation mode. Note though, that the results were only significant at a trend level, so one should be especially cautious when interpreting these findings.

**Table 4:** BIC-scores for the 15 latent class regression models. Models presume three latent classes and differ only in the covariates that are included.

Models	BIC

Empty model (no covariates)	4401
GOSPAN	4408
CRT	4401
Extraversion	4412
Agreeableness	4412
Conscientiousness	4412
Neuroticism	4408
Openness	4409
Social skill	4411
Communication	4410
Imagination	4411
Attention to detail	4407
Attention switching	4411
**RTU**	**4400**
RTC	4408

*Note*. The best fitting model is printed in bold.

**Figure 2:**
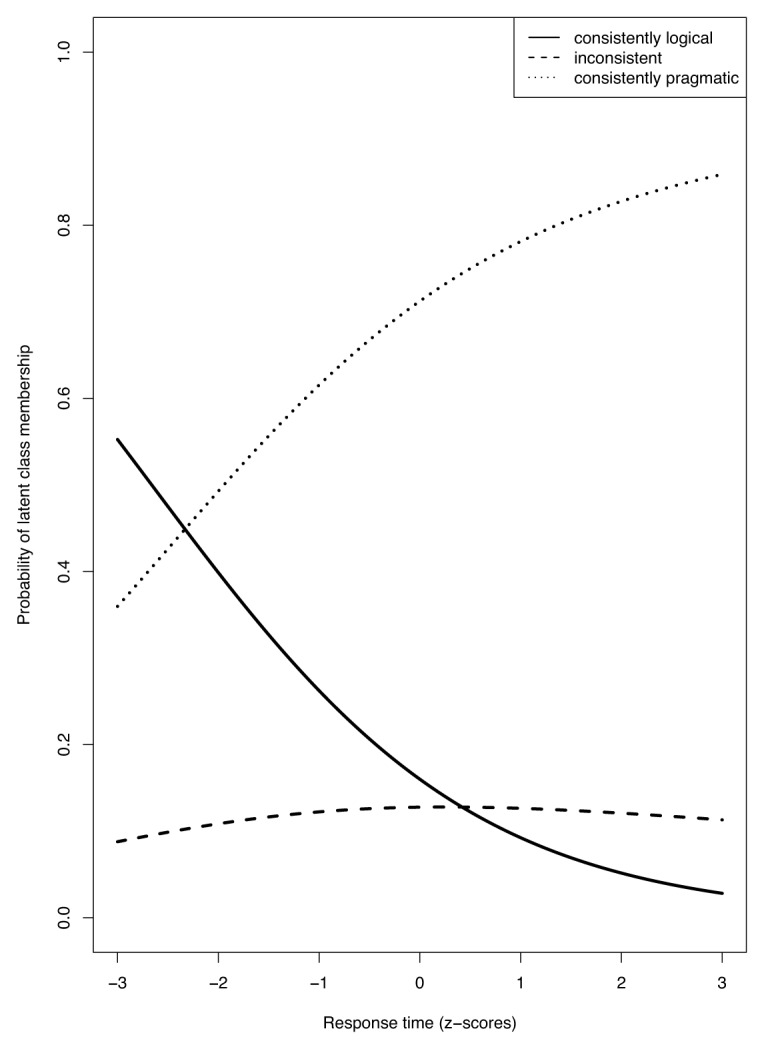
The probability of latent class membership for different response times. The x-axis represents the standardized median response times to the underinformative sentences. The y-axis denotes the predicted probability of latent class membership.

Besides the twelve cognitive and personality covariates, we again also looked at participants’ response times. The results replicated those of Experiment 1 in that response times to the control sentences were unrelated to class membership, while response times to the underinformative sentences did predict membership. Specifically, the probability of belonging to the logical class relative to the pragmatic group increases when Response Time Underinformative decreases (*t*(238) = 2.69, *p* < .01). Likewise, the probability of belonging to the logical group relative to the inconsistent group also increases when Response Time Underinformative decreases, but this result is only significant at a trend level (*t*(238) = 1.77, *p* = .08). There was again no significant difference between the pragmatic class and the inconsistent class (*t*(238) = 0.48, *p* = .63). These findings are in line with those of Experiment 1. They suggest that the slower participants respond to underinformative sentences, the more likely they are to interpret them consistently pragmatic or inconsistent instead of consistently logical (see Figure 2).

In addition to the 14 models that comprised only a single main effect, we also fitted more complex models. Specifically, we evaluated all models that can be formed by combining the 14 main effects. The results showed that neither model provided a better fit than the baseline model, expect for the RTU single main effect model discussed above.

### Discussion

The present experiment sought to examine whether cognitive characteristics (i.e., working memory capacity and the ability to override an intuitive default) and personality traits (i.e., the Big Five dimensions and the subscales of the Autism-Spectrum Quotient questionnaire) are related people’s interpretation of scalar terms. To this end, a latent class analysis was conducted, which a) showed again that there are three interpretation modes (i.e., logical, pragmatic and inconsistent) and b) revealed that neither the cognitive nor the personality covariates were predictive for class membership. However, participants’ response times to the underinformative sentences did relate to interpretation mode, thereby replicating the findings from Experiment 1. That is, the faster participants responded to such sentences, the more likely they were to consistently endorse a logical interpretation instead of an inconsistent or a consistent pragmatic interpretation.

The fact that none of the included variables, except response times to underinformative sentences, could (partly) explain the interindividual variability in interpretation mode was somewhat surprising. Recall that three of the covariates were included to test specific hypotheses derived from the literature (i.e., working memory capacity, the ability to override a default, and communicative skills), while the others were merely added for exploratory purposes. Hence, in what follows, we will focus on the former variables and discuss the theoretical implications of the present findings.

To reiterate, there are different views on how people arrive at the pragmatic *not all* interpretation of *some*. Default theories assume that this enrichment occurs automatically, whereas relevance theory and two-step models posit that it requires cognitive resources. Based on the latter proposition one could predict that people with limited working memory capacity endorse the effortful pragmatic reading to a lesser extent because they lack the necessary resources. However, such a claim is not supported by the data as working memory capacity was not related to interpretation mode. This is in line with results of Dieussaert et al., (2011), who found no effect of working memory capacity on the number of pragmatic responses, but it contrasts with the negative correlation reported by Feeney and colleagues (2004). At first glance these findings seem incompatible with the notion that a pragmatic understanding is cognitively demanding. On the other hand, one could argue that the cognitive cost involved in a pragmatic interpretation is relatively low such that even individuals with limited working memory capacity routinely move to an enriched interpretation. There is some evidence in support of the latter assumption. De Neys and Schaeken (2007) found that a logical reading becomes more prevalent when participants have to remember a complex dot pattern, which indicates that a pragmatic interpretation is not automatic (see also Marty & Chemla, 2013). Critically, Dieussaert and colleagues showed that this logical shift under high load is only true for people with low working memory scores. In other words, limited working memory capacity in itself does not lead to fewer pragmatic interpretations unless an additional cognitive load is imposed.

A similar hypothesis was advanced regarding people’s ability to overcome a default (measured by the CRT). That is, those who are able to refute the (erroneous) intuitive answer to a math puzzle, may also be more inclined to move to a more cognitively demanding interpretation of a scalar term. However, the results did not show a pragmatic to logical shift, or vice versa. If anything, the results suggested that participants tended to be more consistent in their interpretation when they scored high on the CRT. It seems that people who are less prone to use heuristics are more likely to be persistent in their interpretation of underinformative sentences.

Finally, based on findings from Nieuwland et al. (2010) it was predicted that communicatively skilled individuals would be more likely to adopt a pragmatic interpretation mode. Yet, the present results did not find a relation between interpretation mode and scores on the Communication subscale of the AQ questionnaire. The behavioral data of this experiment thus do not converge with the neurological pattern observed by Nieuwland and colleagues. However, the results concur with behavioral studies comparing people with autism spectrum disorders to high-functioning controls (Chevallier, Wilson, Happé, & Noveck, 2010; Pijnacker, Hagoort, Buitelaar, Teunisse, & Geurts, 2009). It is not uncommon though that behavioral markers yield different results than N400 data (Kutas & Federmeier, 2011). Indeed, it is precisely the strength of ERP’s to provide insight into aspects of cognition that are impenetrable with behavioral measures. Taken together, the findings thus far seem to indicate that communicatively skilled people exhibit a different neurological response to pragmatic violations, but that this does not necessarily result in more pragmatic interpretations of underinformative sentences.

## General discussion

The present article examined people’s understanding of scalar terms using latent class analysis. It demonstrated that one can adopt three different interpretation modes when confronted with underinformative sentences such as ‘*Some oaks are trees’*. That is, people either consistently endorse the pragmatic *not all* interpretation, or they consistently hold the logical *some and possibly all* interpretation, or they change their interpretation over sentences. Furthermore, we examined whether this interindividual variability could be explained by text characteristics (i.e., font size, which was manipulated in Experiment 1), response times (Experiment 1 and 2), personality traits (i.e., the Big Five dimensions and the subscales of the AQ questionnaire, Experiment 2), and cognitive abilities (i.e., working memory capacity and the ability to override an intuitive default, Experiment 2). The results showed that only response times to underinformative sentences were reliably related class membership. The remainder of the discussion will first elaborate on the role of response times and then address why the other variables were not predictive for class membership.

Across two experiments, it was shown that the faster people respond, the more likely they are to interpret *some* consistently logical as opposed to consistently pragmatic or inconsistent. The latter should not be taken as a causal statement (i.e., “being a slow reader/responder causes one to adopt a pragmatic interpretation”). For one because response times to control sentences were not predictive for class membership. Secondly, response times to underinformative sentences can be considered as a proxy for the cognitive costs involved to endorse a certain interpretation. This would reverse the causality (i.e., a pragmatic reading of *some* causes one to be a slow responder, presumably because the derivation is effortful). The latter explanation, which has been advanced by Bott and Noveck (2004), is incompatible with default theories as they consider the pragmatic interpretation to be automatic and effortless.

The finding that a pragmatic interpretation of an underinformative sentence requires more time has been replicated in several studies (e.g., Bott et al., 2012; Tomlinson et al., 2013). Several theories have been proposed to account for this effect (i.a., relevance theory, constraint-based one-step models, two-step models, the grammatical account,…), but two critical questions still remained unanswered: (a) how general is this effect and (b) does the additional processing time associated with a pragmatic reading of underinformative sentences actually imply that the scalar inference itself is costly? We will consider both these issues starting with (b).

According to Bott and colleagues (2012), the delayed pragmatic interpretation of an underinformative sentence is at least partly the result of a cognitively demanding derivation process. This assertion is supported by a recent study by Tomlinson and colleagues (2013), which used a cursor movement tracking technique. Specifically, participants performed a sentence verification task by clicking on one of two response boxes labelled “true” and “false”. The response boxes were presented in opposite corners at the top of the screen and the cursor was initially located at the bottom center of the screen. Participants responding “false” to underinformative sentences first tended to drift towards the “true” box before eventually converging on the “false” button, whereas participants responding “true” more or less followed a straight line. Nevertheless, the assumption that the scalar inference itself is cognitively costly has been challenged by Marty and Chemla (2013). These authors argued that the *decision* to enrich the interpretation of the scalar term requires cognitive resources rather than the derivation per se.

The second debated issue concerns the generality of the effect. That is, several studies, using other paradigms than the verification of underinformative sentences, raised questions as to whether scalar inferences are *in general* cognitively costly. Breheny, Katsos, and Williams (2006) reported that participants in a self-paced reading task spend more time processing fragments that contained a scalar term when the context invited an enriched interpretation (e.g., “Mary asked John whether he intended to host all his relatives in his tiny apartment. John replied that he intended to host some of his relatives.”). However, a recent carefully controlled study by Politzer-Ahles and Fiorentino (2013) did not replicate this effect. Furthermore, two studies using the visual world eye-tracking paradigm provided mixed evidence regarding the time course of scalar inferences. Where Grodner, Klein, Carbary, and Tanenhaus (2010) report that it is computed immediately, Huang and Snedeker (2009) found a short delay in the computation of the scalar inference. Note also that pragmatic derivations other than scalar inferences are not associated with slower reading times. That is, people understand idioms like “the cat is out of the bag” or sarcasm as fast as literal utterances (Gibbs, 1986; Ortony, Schallert, Reynolds, & Antos, 1978). Taken together, even though sentence verification data in this and other studies (Bott & Noveck, 2004; Bott et al., 2012; Tomlinson et al., 2013) clearly demonstrate that pragmatic interpretations of underinformative sentences are slower, it does not entail that scalar inferences are in general cognitively costly.

Besides response time to underinfomative sentences no other variable was related to class membership. Nevertheless, these null results have some theoretical and methodological implications. First of all, the font wherein the sentences were presented was manipulated in Experiment 1 because hard to read fonts are argued to trigger more elaborate processing, which should reduce the reliance on defaults (Alter et al., 2007). As explained in the introduction, there are different views on how scalar inferences arise. More specifically, some theories argue that a scalar term elicits a default interpretation, which can be cancelled or enriched (i.e., default theories and two-step models). Hence, these theories might predict that a font manipulation would influence participants’ interpretation of underinformative sentences, which was not the case. At first glance, one could interpret this as evidence for non-default theories (i.e., relevance theory, constraint-based one-step models), yet there are two caveats. For one, Alter and colleagues used the font manipulation to examine high-order cognitive processes involved in syllogistic reasoning and solving math problems. It is entirely possible that these effects do not translate to interpretations of scalar terms. Our failure to obtain an effect of font on participants’ response times and the reanalysis of the data from Reber and Schwarz (1999) cast some doubt on the generalizability of the perceptual fluency effect. The latter study also manipulated sentence visibility by changing the font and examined the effect on truth value judgments. More specifically, they looked at evaluations of sentences like ‘*Osorno is in Chile*’, which did not contain scalar terms. Reber and Schwarz reported that highly visible statements were more likely to be judged as true in comparison to moderately visible statements. Yet the statistical evidence was weak and a Bayesian reanalysis of their results actually supported the null hypothesis (i.e., no effect of sentence visibility). For these reasons, we do not believe that the null effect of font observed in our study provides strong evidence for non-default models.

Secondly, neither working memory capacity nor people’s ability to override a default predicted class membership. Taken together, these findings seem to indicate that the cognitive costs involved in a pragmatic interpretation (predicted by contextual accounts and two-step models) or in cancelling a pragmatic reading and moving to a logical interpretation (predicted by default accounts) are limited. This explanation is intuitively compelling as it would be unfeasible to frequently draw a cognitively demanding inference. It is especially true from a contextualist point of view as a pragmatic understanding of *some* is so pervasive that a burdensome derivation would be unparsimonious. The finding that cognitive skills were not predictive for class membership corroborates conclusions from developmental studies. For instance, Antoniou, Grohmann, Kambanaros, and Katsos (2013) report that 6-to 12-year old children’s ability to comprehend implicatures was unrelated to a range of cognitive factors. These findings suggest that the interpretation of scalar terms throughout the lifespan is unrelated to working memory capacity and other cognitive skills. Instead, it has been suggested that *linguistic* skills play a major role in the development of pragmatic competence (Katsos, Andrés Roqueta, Estevan, & Cummins, 2011). This assertion is supported by the observation that children with Specific Language Impairment, which manifests itself as a delay in the development of receptive and/or expressive language, have an impaired understanding of scalar terms (Katsos et al., 2011). Yet, the present study found no link between a number of personality covariates, including communicative ability, and interpretation mode. Although communicative ability is arguably only one facet of linguistic skill, previous work from Nieuwland and colleagues (2010) did show a relation between communicative ability, measured by the Communication subscale of the AQ questionnaire, and neurological responses to underinformative sentences. This finding supports the idea that pragmatically skilled people are more sensitive to underinformative utterances. However, the present failure to replicate this finding on a behavioral level might indicate that sentence verification data are not ideal to detect such subtle effects (see also Chevallier et al., 2010; Pijnacker et al., 2009) or that our sample was too homogeneous in terms of communicative ability.

Taken together, the present article sought to uncover systematic interindividual differences in people’s understanding of scalar terms. The literature on scalar inferences has mostly focused on the underlying processes, thereby generally ignoring interindividual differences. Specifically, many studies have investigated whether a certain manipulation elicits more pragmatic (or logical) interpretations, while the main goal of this research was to provide more insight as to why people *vary* in their interpretation of scalar terms. To this end, we used latent class regression, which is a viable alternative to analyze data from a typical scalar implicature experiment. The conventional data analysis involves calculating the percentage pragmatic answers for every participant over all (underinformative) items and examining whether the manipulation has an effect on these percentages. A peculiarity here is that the data form a U-shaped distribution. The reason is that a participant often interprets (almost) *all* underinformative sentences pragmatically or logically, resulting in, respectively, an extremely high percentage pragmatic answers or an extremely low percentage. Since these data severely violate the normality assumption, several traditional statistical tests like ANOVA and the Student’s t-test are ineligible. Latent class regression analysis circumvents this issue and it also allows us to add covariates to predict class membership.

In sum, the results of this study showed that the response time to underinformative sentences was a reliable predictor for class membership, but no other variables (i.e., working memory capacity, non-linguistic cognitive skills, and personality traits) were related to participants’ interpretation. As to why people differ in their understanding of scalar terms thus remains an open question. However, the present research provides a starting point for future research as it eliminates some explanations by using a sound statistical framework.

## Footnotes

Due to an inattention, the number of participants in both conditions was not perfectly balanced. There were 90 participants in the normal font condition and 49 in the hard to read font condition.

A Bayesian two sample t-test (Rouder, Speckman, Sun, Morey & Iverson, 2009) favors the null hypothesis (i.e., response times in both font conditions are equal), though the evidence is weak (BF = 3.43, with a Cauchy distribution on effect size).

Even though constraint-based one-step models can account for findings indicating that the scalar inference is cognitively costly (e.g., Tomlinson et al., 2013), they do not consider this to be a general phenomenon. That is, the derivation that *some* means *not all* can be relatively rapid and less demanding in certain situations (Degen & Tanenhaus, 2014).
